# Intraoperative Transfusion of Fresh Frozen Plasma Predicts Morbidity Following Partial Liver Resection for Hepatocellular Carcinoma

**DOI:** 10.1007/s11605-020-04652-0

**Published:** 2020-06-03

**Authors:** Jan Bednarsch, Zoltan Czigany, Isabella Lurje, Christian Trautwein, Tom Lüdde, Pavel Strnad, Nadine Therese Gaisa, Alexandra Barabasch, Philipp Bruners, Tom Ulmer, Sven Arke Lang, Ulf Peter Neumann, Georg Lurje

**Affiliations:** 1 Department of Surgery and Transplantation, University Hospital RWTH Aachen, Aachen, Germany; 2 Department of Medicine III, University Hospital RWTH Aachen, Aachen, Germany; 3 Institute of Pathology, University Hospital RWTH Aachen, Aachen, Germany; 4 Department of Radiology, University Hospital RWTH Aachen, Aachen, Germany; 5 Department of Surgery, Maastricht University Medical Centre (MUMC), Maastricht, Netherlands; 6 Department of Surgery, Campus Charité Mitte | Campus Virchow-Klinikum, Charité-Universitätsmedizin Berlin, Augustenburger Platz 1, 13353 Berlin, Germany

**Keywords:** Hepatocellular carcinoma (HCC), Fresh frozen plasma (FFP), Perioperative morbidity

## Abstract

**Background:**

The reduction of perioperative morbidity is a main surgical goal in patients undergoing partial hepatectomy for hepatocellular carcinoma (HCC). Here, we investigated clinical determinants of perioperative morbidity in a European cohort of patients undergoing surgical resection for HCC.

**Methods:**

A total 136 patients who underwent partial hepatectomy for HCC between 2011 and 2017 at our institution were included in this analysis. The associations between major surgical complications (Clavien-Dindo ≥ 3) and overall morbidity (Clavien-Dindo ≥ 1) with clinical variables were assessed using univariate and multivariable binary logistic regression analysis.

**Results:**

Multivariable analysis identified the Child-Pugh-Score (CPS, HR = 3.23; *p* = 0.040), operative time (HR = 5.63; *p* = 0.003), and intraoperatively administered fresh frozen plasma (FFP, HR = 5.62; *p* = 0.001) as independent prognostic markers of major surgical complications, while only FFP (HR = 6.52; *p* = 0.001) was associated with morbidity in the multivariable analysis. The transfusion of FFP was not associated with perioperative liver functions tests.

**Conclusions:**

The intraoperative administration of FFP is an important independent predictor of perioperative morbidity in patients undergoing partial hepatectomy for HCC.

**Electronic supplementary material:**

The online version of this article (10.1007/s11605-020-04652-0) contains supplementary material, which is available to authorized users.

## Introduction

Hepatocellular carcinoma (HCC) is the fifth most common malignancy worldwide and its mortality ranks third among all solid tumors, behind only carcinomas of the lung and the colon.^1^,^2^ Due to the underlying chronic liver disease in most patients with HCC, orthotopic liver transplantation (OLT) is considered the treatment of choice since it treats both underlying liver disease and the malignant tumor. However, liver resections are increasingly utilized, since the availability of liver allografts remains low in many developed countries.^3^–^5^ Correspondingly, a number of studies demonstrated that partial hepatectomy can be carried out safely even in patients with advanced liver disease.^6^–^8^ This development is based on an improved preoperative assessment of patient-related risk factors for dismal operative outcome and the introduction of novel surgical techniques. Recent advancements in the modern era of HCC-surgery include several strategies for the dynamic assessment of the liver function and the implementation of advanced laparoscopic liver surgery.^9^,^10^ While the latter resulted in comparable oncological results with reduced intraoperative blood loss and improved postoperative recovery, dynamic liver function tests such as the LiMAx (maximum liver function capacity), indocyanine green, and the hepato-imino diacetic acid (HIDA) test^9^,^11^–^13^ provide a dynamic estimate of the functional liver reserve and improve the preoperative risk evaluation in these patients.^14^,^15^

Even though partial liver resection for HCC has developed into a safe and reasonable approach in these patients, the ideal management of surgical morbidity remains subject of ongoing debate.^16^ Recent data show the prognostic significance of perioperative complications in terms of oncologic outcomes such as disease-free (DFS) and overall survival (OS) in HCC.^17^–^19^

Here, we aim to investigate clinical factors associated with perioperative morbidity in a European cohort of patients undergoing surgical resection for HCC.

## Patients and Methods

### Patients

Between 2011 and 2017, one hundred thirty-six (*n* = 136) patients with HCC who were treated with surgical resection at the University Hospital RWTH Aachen (UH-RWTH) were included in this study. All of these patients had localized tumors without signs of systemic disease. Clinical staging was performed according to the International Union Against Cancer (UICC) criteria. The study was conducted at the UH-RWTH in accordance with the requirements of the Institutional Review Board of the RWTH-Aachen University (EK 360/15), the current version of the Declaration of Helsinki, and the good clinical practice guidelines (ICH-GCP).

### Staging and Surgical Technique

All patients who were referred for surgical treatment to our institution underwent a detailed clinical work-up as previously described.^2^,^20^,^21^ This included the assessment of the number, size, and location of tumor nodules as well as the presence of distant metastases by cross-sectional imaging (gadolinium-based magnetic resonance imaging (MRI) or contrast-material enhanced computed tomography (cmCT)). The patients’ perioperative risk was determined based on the American society of anesthesiologists- (ASA) and the Eastern Cooperative Oncology Group (ECOG)-performance status, CT or MRI-based 3D-calculation of the future liver remnant (FLR), as well as the analysis of the quantitative and functional parenchymal liver function as assessed by laboratory parameters and the LiMAx test (Humedics® GmbH, Berlin, Germany).^16^ The decision for hepatectomy as the primary treatment for the individual patient was made by a staff hepatobiliary surgeon and was approved by the institutional interdisciplinary tumor board in all cases. Patients staged BCLC A to BCLC C without any evidence of extrahepatic spread as well as compensated liver function were considered candidates for surgical therapy. Liver resection was carried out in accordance with common clinical standards. An intraoperative ultrasound was performed to visualize the local tumor spread and other suspicious lesions. The decision for either anatomic resections—defined by resection of the related portal vein branch—or non-anatomic atypical wedge resections with an adequate resection margin was based on the surgeon’s preference. In general, non-anatomic atypical wedge resections were preferred for small, peripherally located, and solitary HCCs that exhibited exophytic growth.

Blood parameters associated with coagulation and transfusion requirement were assessed 1 day prior to surgery in all patients. No FFP or any other blood product was applied prophylactically prior to surgery. The anesthesiologic management comprised restrictive fluid intervention strategy aiming to maintain a low central venous pressure (CVP) during parenchymal dissection. This was further facilitated by reverse Trendelenburg positioning or veno-dilative medication if necessary. During surgery, arterial blood gas analyses were regularly conducted to measure hemoglobin and lactate, while thromboelastography (TEG) was carried out on demand. Parenchymal transection was carried out using the Cavitron Ultrasonic Surgical Aspirator (CUSA®, Integra LifeSciences®, Plainsboro NJ, USA) with low CVP and intermittent Pringle maneuvers if necessary. Intraoperative blood transfusions were administered based on a restrictive transfusion policy based on an interdisciplinary case-by-case decision between surgeon and anesthesiologist with respect to the amount of blood loss, results of the regularly obtained blood gas analyses and individual co-morbidities. A target hemoglobin concentration of 7–9 g/dl was maintained during surgery, and FFPs were administered on a case-by-case decision in cases of coagulopathic bleeding. Platelet transfusions were administered only in cases of pathological TEG results.

The patients were directly transferred to a specialized intensive care unit (ICU) after the procedure. The transfer to a normal postoperative ward was usually carried out on the 1st postoperative day (POD). Patients were later released from the hospital following a final evaluation by the attending surgeon.

All specimens were evaluated for tumor size, histological diagnosis, tumor grading, tumor staging, vessel invasion, resection margin, and presence of cirrhosis by an experienced staff pathologist.

### Statistical Analysis

The primary endpoint of this study was major perioperative inhouse morbidity in HCC patients undergoing surgical resection in curative intent, which was defined as complications rated Clavien-Dindo ≥ 3 according to the Clavien-Dindo scale.^22^ The secondary endpoint was the presence of any postoperative complication during hospitalization (Clavien-Dindo ≥ 1).^22^ All complications are reported as inhouse morbidity. Data derived from continuous variables are presented as mean and standard deviation. Associations between perioperative variables and the primary or secondary endpoint were assessed by means of binary logistic regression. Variables showing a *p* value < 0.1 in univariate analysis were transferred into a multivariable model and analyzed with multivariable binary logistic regressions using backward elimination. For this purpose, nominal and categorical data were recoded into a scaled dummy variable. Survival curves were generated by the Kaplan-Meier method under exclusion of perioperative mortality and compared with the log-rank test. Median follow up was accessed with the reverse Kaplan-Meier method. The level of significance was set to *p* < 0.05, and *p* values are given for two-sided testing. Analyses were performed using SPSS Statistics 24 (IBM Corp., Armonk, NY, USA).

## Results

### Preoperative, Operative and Postoperative Data

A total of 136 patients with a mean age of 67 ± 12 years and mean body mass index (BMI) of 27 ± 5 kg/m^2^ underwent curative surgery for HCC at our institution from 2011 to 2017. More than half of the patients (61.1%, 83/136) had a preoperative performance status ASA III or higher assessed by the attending anesthesiologist. The majority of the patients were classified as Child Pugh A (89.7%, 122/133) with a mean Child Pugh Score (CPS) of 5.3 ± 0.7 and a mean model for end-stage liver disease (MELD) of 7 ± 3. Milan criteria was fulfilled in 40 patients (29.4%). One fifth of the patients (19.9%, 27/136) underwent laparoscopic liver resection, and mean operative time was 210 ± 83 min. During surgery, packed red blood cells (RPC) were administered to 33 patients (27.3%) and FFP to 51 patients (42.1%). The cohort had a mean hospital stay of 14 ± 13 days after surgery. Approximately 45% (62/136) showed no postoperative complications while 40 patients (29.4%) experienced major postoperative complications (Clavien-Dindo ≥ 3). The mean postoperative comprehensive complication index (CCI) was 20 ± 29. Detailed clinicopathological and perioperative characteristics are outlined in Table 1. Additionally, a concise overview of postoperative complications is presented in Table 2.

**Table 1:** Clinical and perioperative characteristics

Demographics	mean ± SD
Gender, m/f (%)	96 (67.6)/40 (29.7)
Age (years)	67 ± 12
BMI (kg/m^2^)	27 ± 5
Portal vein embolization, *n* (%)	7 (6.6)
ASA, *n* (%)	
I	0
II	53 (38.9)
III	78 (57.4)
IV	5 (3.7)
V	0
Milan criteria, *n* (%)	40 (29.4)
BCLC, *n* (%)	
0	3 (2.2)
A	77 (56.6)
B	33 (24.3)
C	19 (14.0)
D	0
Preoperative liver function	Mean ± SD
MELD Score	7 ± 3
Albumin (g/dl)	39 ± 7
AST (U/l)	56 ± 44
ALT (U/l)	48 ± 42
GGT (U/l)	172 ± 173
Total bilirubin (mg/dl)	0.6 ± 0.4
Platelet count (/nl)	239 ± 112
Alkaline Phosphatase (U/l)	123 ± 90
Prothrombine time (%)	93 ± 14
INR	1.04 ± 0.10
Creatinine (mg/dl)	1.0 ± 0.8
Hemoglobin (g/dl)	13.1 ± 1.9
Child Pugh, *n* (%)	
A	122 (89.7)
B	11 (8.1)
C	0
Child Pugh score	5.3 ± 0.7
Operative data	Mean ± SD
Laparoscopic resection, n (%)	27 (19.9)
Conversation rate, n (%)	3 (10)
Conversion due to bleeding, n (%)	2 (66.7)
Operative time (minutes)	210 ± 83
Operative procedure, n (%)	
Atypical	39 (28.7)
Segmentectomy	13 (16.9)
Bisegmentectomy	12 (8.8)
Hemihepatectomy	34 (25.0)
Extended liver resection	23 (16.9)
other	5 (3.7)
Additional procedures (RFA, etc.), *n* (%)	3 (2.2)
Pringle maneuver, *n* (%)	7 (5.2)
Duration of Pringle maneuver (min)*	20 (15–25)
Intraoperative blood transfusion, *n* (%)	33 (27.3)
Intraoperative FFP, *n* (%)	51 (42.1)
Intraoperative platelet transfusion, *n* (%)	3 (2.5)
Pathological examination	mean ± SD
R0 resection, *n* (%)	127 (93.4)
Largest tumor diameter (mm)	67 ± 41
Number of nodules	1.9 ± 1.4
Macrovascular invasion, *n* (%)	32 (23.5)
Tumor stage UICC, *n* (%)	
I	48 (35.3)
II	49 (36.0)
IIIA	21 (15.4)
IIIB	8 (5.9)
IIIC	2 (1.5)
IVA	3 (2.2)
IVB	1 (0.7)
Postoperative data	mean ± SD
Intensive care stay, days	2 ± 9
Hospitalization, days	14 ± 13
Postoperative complications, n (%)	
No complications	62 (45.6)
Clavien-Dindo I	19 (14.0)
Clavien-Dindo II	15 (11.0)
Clavien-Dindo IIIa	15 (11.0)
Clavien-Dindo IIIb	8 (5.9)
Clavien-Dindo IVa	7 (5.1)
Clavien-Dindo IVb	1 (0.7)
Clavien-Dindo V	9 (6.6)
CCI	20 ± 29
Postoperative liver failure^#^	2 (1.5)
Postoperative blood transfusion	22 (18.2)
Postoperative FFP	9 (7.4)
Postoperative platelet transfusion	5 (4.1)

Data presented as mean and standard deviation if not noted otherwise. *Median and interquartile range. ^*#*^Postoperative liver failure was assessed by the 50–50-criteria.^23^ *ALT,* alanine aminotransferase*; ASA,* American society of anesthesiologists classification*; AST,* aspartate aminotransferase*; BCLC,* Barcelona clinical liver cancer staging system*; BMI,* body mass index*; CCI,* comprehensive complication index*; FFP,* fresh frozen plasma*; GGT,* gamma glutamyltransferase*; INR,* international normalized ratio*; MELD,* model of end stage liver disease*; MWA,* microwave ablation*; UICC,* Union for International Cancer Control

**Table 2:** Detailed overview of postoperative complications

Postoperative complication	*n* (%)
Sub-cohort with intraoperative FFP	
Bile leakage	9 (23.7)
Pneumonia	5 (13.2)
Pleural effusion	4 (10.5)
Ascites	2 (5.3)
Postoperative hemorrhage	2 (5.3)
Surgical site infection	2 (5.3)
Unspecific infection	2 (5.3)
Pulmonary embolism	2 (5.3)
Acute renal failure	2 (5.3)
Cardiac arrhythmia	2 (5.3)
Urinary tract infection	1 (2.6)
Liver insufficiency	1 (2.6)
Portal vein thrombosis	1 (2.6)
Delirium	1 (2.6)
Prolonged postoperative nausea	1 (2.6)
Prolonged postoperative pain	1 (2.6)
Sub-cohort without intraoperative FFP	
Bile leakage	4 (14.8)
Urinary tract infection	3 (11.1)
Ascites	3 (11.1)
Electrolyte disorders	3 (11.1)
Septic shock	2 (7.4)
Postoperative hemorrhage	2 (7.4)
Surgical site infection	1 (3.7)
Pleural effusion	1 (3.7)
Unspecific infection	1 (3.7)
Pulmonary embolism	1 (3.7)
Acute renal failure	1 (3.7)
Pancreatitis	1 (3.7)
Paralytic ileus	1 (3.7)
Pneumothorax	1 (3.7)
Allergic reaction	1 (3.7)
Intraabdominal fluid collection	1 (3.7)

The leading postoperative complication was assessed in every patient who experienced postoperative complications*. FFP,* fresh frozen plasma

### Univariate and Multivariable Analysis of Postoperative Morbidity

A univariate binary logistic regression was carried out for postoperative morbidity (Clavien-Dindo ≥ 1) including all available pre- and intraoperative variables (Table 3). Largest tumor diameter (HR = 2.33; *p* = 0.017), CPS (HR = 2.50; *p* = 0.047), operative time (HR = 2.08; *p* = 0.039), FFP transfusion (HR = 4.39; *p* = 0.001), and laparoscopic resection (HR = 0.41; *p* = 0.047) were associated with postoperative complications (Table 3). Variables showing a *p* value < 0.1 in univariate analysis were included into a multivariable binary logistic regression model which determined FFP (HR = 4.39; *p* = 0.001) as the single significant predictor of postoperative morbidity (Table 4). For major postoperative morbidity (Clavien-Dindo ≥ 3), the univariable analysis showed significant associations of the largest tumor diameter (HR = 2.55; *p* = 0.023), CPS (HR = 2.60; *p* = 0.030), operative time (HR = 3.84; *p* = 0.003), blood transfusions (HR = 2.51; *p* = 0.035), FFP transfusion (HR = 6.52; *p* = 0.001), and major postoperative complications (Table 3). Variables showing a *p* value < 0.1 in univariate analysis were again included in the corresponding multivariable binary logistic regression model which determined CPS (HR = 3.23; *p* = 0.040), operative time (HR = 5.63; p = 0.003) and FFP (HR = 5.62; p = 0.001) as independent predictors of major postoperative morbidity (Table 5).

**Table 3:** Univariable analysis of perioperative morbidity

	*n*	*Major morbidity (Clavien-Dindo ≥ 3)*			*Morbidity (Clavien-Dindo ≥ 1)*		
		Hazard ratio	95% CI	*P* value	Hazard ratio	95% CI	*P* value
Sex				0.467			0.773
Male	96						
Female	40						
Age, years				0.540			0.517
≤ 65	53						
> 65	83						
BMI, kg/m^2^				0.733			0.893
≤ 25	54						
> 25	82						
ASA				0.812			0.865
I/II	62						
III/IV	74						
Milan criteria				0.063			0.172
Yes	40						
No	95						
BCLC Staging				0.587			0.445
0	3						
A	77						
B	33						
C	19						
D	0						
Largest tumor diameter, mm				0.023			0.017
≤ 50	59	1			1		
> 50	76	2.55	1.14–5.69		2.33	1.16–4.67	
Number of nodules				0.427			0.582
Single	84						
Multilocular	49						
Macrovascular invasion				0.443			0.584
No	99						
Yes	32						
MELD				0.130			0.112
≤ 8	61						
> 8	73						
Child Pugh score				0.030			0.047
≤ 5	108	1			1		
> 5	28	2.60	1.10–6.15		2.50	1.01–6.16	
Albumin, g/l				0.579			0.537
≤ 40	46						
> 40	54						
AST, U/l				0.303			0.811
≤ 40	55						
> 40	66						
ALT, U/l				0.598			0.765
≤ 40	57						
> 40	49						
GGT, U/l				0.713			0.486
≤ 100	58						
> 100	62						
Bilirubin, mg/dl				0.826			0.521
≤ 1	61						
> 1	73						
Alkaline phosphatase, U/l				0.508			0.228
≤ 100	60						
> 100	59						
Platelet count, 1/nl				0.152			0.616
≤ 200	54						
> 200	80						
Prothrombin time, %				0.240			0.613
≤ 100	105						
> 100	29						
INR				0.304			0.626
≤ 1	47						
> 1; < 1.2	75						
≥ 1.2	12						
Creatinine, mg/dl				0.952			0.343
< 1	61						
≥ 1	74						
Hemoglobin, g/dl				0.189			0.283
≤ 12	42						
> 12	92						
Operative time, min				0.003			0.039
≤ 180	55	1			1		
> 180	81	3.84	1.60–9.18		2.08	1.04–4.18	
Intraoperative blood transfusion				0.035			0.104
No	88	1					
Yes	33	2.51	1.07–5.87				
Intraoperative FFP				0.001			0.001
No	70	1			1		
Yes	51	6.52	2.68–15.86		4.39	1.99–9.67	
Intraoperative platelet transfusion				0.841			0.666
No	118						
Yes	3						
Laparoscopic resection				0.481			0.047
No	109				1		
Yes	27				0.41	0.17–0.99	
Type of surgery				0.412			0.614
Atypical	39						
Segmentectomy	23						
Bisegmentectomy	12						
Hemihepatectomy	34						
Extended liver resection	23						
other	5						
Pringle maneuver				.417			.630
No	127						
Yes	7						

Various parameters are associated with major and general postoperative morbidity. Hazard ratios are shown for statistically significant variables. *ALT*, alanine aminotransferase; *ASA*, American society of anesthesiologists classification; *AST*, aspartate aminotransferase; *BCLC*, Barcelona clinical liver cancer staging system; *BMI*, body mass index; *FFP*, fresh frozen plasma; *GGT*, gamma glutamyltransferase; *INR*, international normalized ratio; *MELD*, model of end stage liver disease; *UICC*, Union for International Cancer Control. *Mean

**Table 4:** Multivariable binary logistic regression of any perioperative morbidity

Variable	*Morbidity (Clavien-Dindo ≥ 1)*		
	Hazard Ratio	95% CI	*P* value
Largest tumor diameter, mm			0.228
≤ 50	1		
> 50	1.63	0.74–3.60	
Child Pugh score			0.198
≤ 5	1		
> 5	1.96	0.70–5.45	
Operative time, min			0.149
≤ 180	1		
> 180	1.82	0.81–4.10	
FFP			0.001
No	1		
Yes	4.39	1.99–9.67	
Laparoscopic resection			0.213
No	1		
Yes	.549	0.21–1.41	

All variables showing statistical significance in univariate binary logistic regression were included in a multivariable logistic regression. Hazard ratios are shown for statistically significant variables. *FFP*, fresh frozen plasma

**Table 5:** Multivariable binary logistic regression of major perioperative morbidity

Variable	*Morbidity (Clavien Dindo ≥ 3)*		
	Hazard Ratio	95% CI	*P* value
Largest tumor diameter, mm			0.809
≤ 50	1		
> 50	1.20	0.27–5.38	
Child Pugh score			0.040
≤ 5	1		
> 5	3.23	1.06–9.87	
Operative time, min			0.003
≤ 180	1		
> 180	5.63	1.82–17.41	
Blood transfusions			0.395
No	1		
Yes	.608	0.19–1.91	
FFP			0.001
No	1		
Yes	5.62	2.19–14.40	
Milan criteria			0.635
Yes	1		
No	1.51	.28–8.19	

All variables showing statistical significance in univariate binary logistic regression were included in a multivariable logistic regression. Hazard ratios are shown for statistically significant variables*. FFP*, fresh frozen plasma

A similar analysis regarding postoperative and major postoperative morbidity was carried out for the sub-cohort of patients who received intraoperative FFP (*n* = 51). Here, no statistical significance was found except for operative time in association to major postoperative morbidity (HR = 7.33; *p* = 0.006; Supplementary Table S1). Also, we conducted a group comparison regarding operative characteristics of patients with and without intraoperative FFP transfusion. In this analysis, no difference was observed regarding the particular surgical procedure (*p* = 0.305), laparoscopic approach (*p* = 0.135), and the utilization of Pringle maneuver (*p* = 0.111) except for a longer operative time in patients receiving FFP (230 ± 86 min vs. 196 ± 82 min; *p* = 0.027; Supplementary Table S2). No difference was observed in perioperative mortality (7.8%, 4/51 vs. 4.3%, 3/70; *p* = 0.408) and postoperative liver failure (3.9% (2/50) vs. 0/70; *p* = 0.095) between patients with and without intraoperative FFP transfusion.

We further assessed the relationship between intraoperatively transfused FFP and preoperative liver function parameters. Here, no significant association was observed between FFP transfusion and preoperative serum albumin (*p* = 0.671), bilirubin (*p* = 0.767), CPS (*p* = 0.055), International normalized Ratio (INR, *p* = 0.517), MELD (*p* = 0.120), platelet count (*p* = 0.301), and prothrombin time (*p* = 0.392) (Table 6).

**Table 6:** Intraoperatively applied FFP units in relation to liver function parameters

Variable	*FFP units*		
	Mean	Standard deviation	*P* value
Albumin, g/l			0.671
≤ 40	1.98	2.68	
> 40	1.77	2.55	
Bilirubin, mg/dl			0.767
≤ 1	1.89	2.58	
> 1	2.14	2.91	
Child Pugh Score			0.055
≤ 5	1.72	2.51	
> 5	2.80	2.94	
INR			0.517
≤ 1	2.13	2.79	
> 1	1.78	2.50	
MELD			0.120
≤ 8	1.80	2.62	
> 8	2.56	2.53	
Platelet count, 1/nl			0.301
≤ 200	2.26	2.88	
> 200	1.67	2.37	
Prothrombin time, %			0.392
≤ 100	1.80	2.54	
> 100	2.28	2.83	

No statistical difference in intraoperative FFP transfusion between patients grouped by liver function parameters was observed. FFP, fresh frozen plasma; *INR*, international normalized ratio

No statistical associations were observed between intraoperatively and postoperatively applied blood transfusions (*p* = 0.160), FFP (*p* = 0.193), and platelet transfusions (*p* = 0.869).

### Survival Analysis

**Fig. 1:**
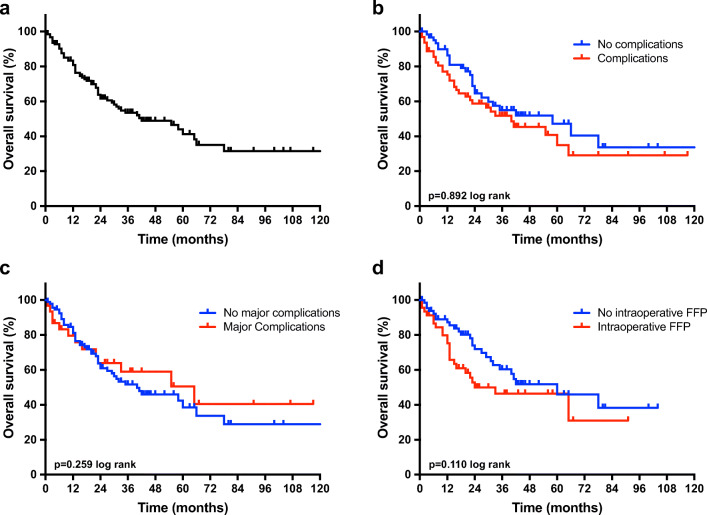
Oncological survival in hepatocellular carcinoma **a** Overall survival in hepatocellular carcinoma. The median OS of the cohort was 3.5 years (95% CI: 1.6–5.4). **b** Overall survival in hepatocellular carcinoma stratified by postoperative complications. The Kaplan-Meier analysis with respect to postoperative complications showed a median OS of 4.8 years (95% CI, 2.2–7.5) in patients without postoperative complications compared to 3.3 years (95% CI, 1.4–5.3) in patients with postoperative complications (*p* = 0.892 log rank). **c** Overall survival in hepatocellular carcinoma stratified by major postoperative complications. The Kaplan-Meier analysis with respect to major postoperative complications (Clavien-Dindo ≥ 3) showed a median OS of 3.3 years (95% CI, 1.4–5.2) in patients without major postoperative complications compared to 5.4 years (95% CI, 1.8–9.0) in patients with major postoperative complications (*p* = 0.259 log rank). **d** Overall survival in hepatocellular carcinoma stratified by intraoperative FFP. The Kaplan-Meier analysis with respect to intraoperative administration of FFP showed a median OS of 5.0 years (95% CI, 2.2–7.8) in patients who have not received intraoperative FFP compared to 2.0 years (95% CI, 0–4.3) in patients who have received intraoperative FFP (*p* = 0.110 log rank). CI, confidence interval; FFP, fresh frozen plasma; OS, overall survival

With a median follow-up of 3.5 years, the OS in our cohort was 3.5 years (95% confidence interval (CI), 1.6–5.4 years). No association was observed between oncological outcome and postoperative morbidity (*p* = 0.345 log rank), major postoperative morbidity (*p* = 0.611 log rank), and intraoperative FFP administration (*p* = 0.110 log rank) (Fig. 1).

## Discussion

The management and prevention of perioperative morbidity have become an important goal in modern-era HCC surgery. Here, we aimed to evaluate the association of various clinicopathological parameters with perioperative outcomes in HCC-patients undergoing surgical resection in curative intent. Our multivariable model identified intraoperative FFP transfusion as an independent prognostic marker for overall morbidity and intraoperative FFP transfusion, CPS, and operative time as independent predictors for major morbidity.

The adverse effects of blood products on surgical and oncological outcomes in HCC have been demonstrated earlier.^24^–^26^ It is generally assumed that blood transfusions adversely affect long-term outcomes, especially in patients with early-stage HCCs.^27^–^29^ While the effects of blood transfusions in liver surgery have been investigated before, the exact significance of FFP transfusion in clinical outcomes in HCC patients undergoing partial hepatectomy in curative intent remains to be determined.^30^ Historically, Japanese centers advocate a relatively liberal usage of FFPs compared with European and North-American centers, which propagate more restrictive administration of FFPs in this setting.^31^ Tomimaru et al. compared long-term outcome of 297 patients with FFP transfusion on demand with a historical cohort of 204 patients which routinely received FFP postoperatively and found no difference in oncologic outcome.^32^ A further Japanese report also concluded that the administration of FFP—in contrast to RPC—does not influence survival of patients undergoing partial hepatectomy for HCC.^33^ Although FFPs have already shown an adverse effect on oncologic outcome in other malignancies (pancreatic cancer, colorectal liver metastases), clinical evidence suggesting a similar effect in HCC is lacking.^34^,^35^

Shiba et al. assessed the perioperative complications in 99 patients undergoing hepatectomy for HCC. In this cohort, pulmonary complications were associated with the cumulative count of perioperatively transfused FFPs in multivariable analysis. Unlike our study, Shiba et al. evaluated the role of cumulatively transfused FFPs in both the intra- and postoperative course.^36^ Therefore, the present study is the first to report on the adverse effects of intraoperatively transfused FFPs in terms of perioperative outcome in patients with HCC. The requirement of postoperative FFPs administration is commonly attributed to the lack of clotting factors in association with a postoperative liver dysfunction. Moreover, liver failure per se is associated with an increased rate of bacterial infections, and the onset of hepatopulmonary syndrome might also influence the rate of pulmonary complications.^37^ Therefore, postoperative FFP transfusion is mostly required in cases with significant postoperative liver dysfunction/failure which might explain the observed predisposition to postoperative complications in the above-mentioned study of Shiba et al. This fact makes it difficult to interpret a causal relationship between postoperative FFP administration and perioperative outcomes.

Clot formation can be facilitated by the maintenance of core body temperature > 35 °C, pH > 7.2 and plasma calcium levels > 1 mmol/L.^38^ From a surgical point of view, transfusion requirements can also be reduced by surgical technique and the adherence to low CVP by restrictive transfusion and volume administration policy during parenchymal dissection.^39^ It is reasonable to assume that the need of intraoperative transfusion may also be associated with the underlying chronic liver disease. In our study, the preoperative assessment of the liver function was not associated with postoperative morbidity. Also, we did not find a relationship between preoperative liver function and intraoperatively transfused FFPs, highlighting the importance of an optimized intraoperative blood management (Table 6).

The association of FFP transfusion with perioperative morbidity in partial hepatectomy for HCC might be explained with the effect of transfusion-related immunomodulation. As such, Sarani et al. found a correlation between transfusion of FFP and pulmonary or blood stream infections in critically ill surgical patients.^40^ Some investigators speculated that soluble proteins in FFP may cause similar immunosuppressive effects as seen with RBC transfusions.^40^ Such proteins may include human leukocyte antigen and fibrinogen/fibrin degradation products or disrupted white blood cell products. These components can be found in FFPs, even after leukoreduction and may alter the immune response.^41^ Discussed mechanisms include diminished antigen processing by macrophages, upregulation of both T suppressor and regulatory cells and humoral immunosuppressive mediators, impaired natural killer cell activity, and production of anti-idiotypic antibodies.^41^ However, there is no definitive or satisfactory explanation for transfusion-related immunomodulation and many pathophysiological aspects of this phenomenon remain to be determined. Interestingly, the transfusion-related immunomodulation and the discussed pathomechanisms are also linked to increased rates of cancer recurrence.^41^ Thus, future studies should explore the association between FFP and oncologic outcome as it has already been demonstrated for other malignancies.^34^,^35^

Like any other perioperative outcome study, our analysis has certain inherent limitations. All patients included in this study were treated at a single institution reflecting our local clinical approach and the study is based on retrospective data which were not obtained in the setting of a controlled prospective clinical trial. As such, our transfusion strategy did not follow a strict study protocol. Further, our sample size is relatively small compared to some other studies especially from Asian cohorts. Therefore, further subset analyses of patients who received intraoperative FFPs were not possible. However, due to the lower incidence of HCC in Europe, European studies with significantly larger sample sizes are mostly multi-center analyses with different clinical approaches to this complex disease, while all patients of our cohort were treated according to the same clinical standards. Also, in eastern and western specialized centers, HCC patients present with different epidemiologic and clinical characteristics, including tumor biology and clinical behavior, necessitating slightly different therapeutic approaches. Thus, results and conclusions from Asian cohorts might not be applicable to Western patients.^42^,^43^

Notwithstanding the aforementioned limitations, we have identified intraoperative administration of FFPs as an important predictor of perioperative morbidity in patients undergoing liver resections for HCC. A more restrictive policy in terms of intraoperative FFP transfusions by optimizing surgical and anesthesiologic conditions should be a key goal in partial hepatectomy for HCC. Larger, prospective clinical trials are needed to confirm and validate our findings in HCC and other hepatic malignancies.

## Electronic supplementary material

ESM 1 (DOCX 64 kb)

ESM 2 (DOCX 19 kb)

## Abbreviations

ALT: Alanine aminotransferase
AP: Alkaline phosphatase
ASA: American society of anesthesiologists
AST: Aspartate aminotransferase
BMI: Body mass index
CCI: Comprehensive complication index
CPS: Child Pugh Score
CI: Confidence interval
CRP: C-reactive protein
cmCT: Contrast material enhanced computed tomography
CUSA: Cavitron Ultrasonic Surgical Aspirator
CVP: Central venous pressure
DFS: Disease-free survival
ECOG: Eastern Cooperative Oncology Group
FFP: Fresh frozen plasma
FLR: Future liver remnant
GCP: Good clinical practice
GGT: Gamma glutamyltransferase
HCC: Hepatocellular carcinoma
HIDA: Hepato-imino diacetic acid
INR: International normalized ratio
LiMAx: Maximum liver function capacity
MELD: Model of end-stage liver disease
MRI: Magnetic resonance imaging
OLT: Orthotopic liver transplantation
OS: Overall survival
POD: Postoperative day
TEG: Thromboelastography
UH-RWTH: University Hospital Rheinisch-Westfälische Technische Hochschule
UICC: Union for international cancer control
