# Supplementary material for: Intraoperative Transfusion of Fresh Frozen Plasma Predicts Morbidity Following Partial Liver Resection for Hepatocellular Carcinoma
Source: J Gastrointest Surg. 2020 Jun 3;25(5):1212–23. doi: 10.1007/s11605-020-04652-0 (PMC8096754; doi:10.1007/s11605-020-04652-0)
Supplement: Supplementary file 1 — (DOCX 64 kb) [file 11605_2020_4652_MOESM1_ESM.docx]

**Supplementary Table S1:** Univariable analysis of perioperative morbidity in patients who received intraoperative FFP

|  | **n** | ***Major morbidity (Clavien Dindo ≥ 3)*** | | |  | ***Morbidity (Clavien Dindo ≥ 1)*** | | |
| --- | --- | --- | --- | --- | --- | --- | --- | --- |
|  |  | **Hazard Ratio** | **95% CI** | ***P* value** |  | **Hazard Ratio** | **95% CI** | ***P* value** |
| **Sex** |  |  |  | .381 |  |  |  | .614 |
| Male | 38 |  |  |  |  |  |  |  |
| Female | 13 |  |  |  |  |  |  |  |
| **Age, years** |  |  |  | .925 |  |  |  | .957 |
| ≤ 65 | 16 |  |  |  |  |  |  |  |
| > 65 | 35 |  |  |  |  |  |  |  |
| **BMI, kg/m^2^** |  |  |  | .856 |  |  |  | .444 |
| ≤ 25 | 19 |  |  |  |  |  |  |  |
| > 25 | 32 |  |  |  |  |  |  |  |
| **ASA** |  |  |  | .658 |  |  |  | .694 |
| I / II | 22 |  |  |  |  |  |  |  |
| III / IV | 29 |  |  |  |  |  |  |  |
| **Milan criteria** |  |  |  | .811 |  |  |  | .341 |
| Yes | 13 |  |  |  |  |  |  |  |
| No | 38 |  |  |  |  |  |  |  |
| **BCLC Staging** |  |  |  | .939 |  |  |  | .806 |
| 0 |  |  |  |  |  |  |  |  |
| A | 29 |  |  |  |  |  |  |  |
| B | 10 |  |  |  |  |  |  |  |
| C | 11 |  |  |  |  |  |  |  |
| D |  |  |  |  |  |  |  |  |
| **Largest tumor diameter, mm** |  |  |  | .843 |  |  |  | .368 |
| ≤ 50 | 17 |  |  |  |  |  |  |  |
| > 50 | 34 |  |  |  |  |  |  |  |
| **Number of nodules** |  |  |  | .910 |  |  |  | .681 |
| Single | 31 |  |  |  |  |  |  |  |
| Multilocular | 20 |  |  |  |  |  |  |  |
| **Macrovascular Invasion** |  |  |  | .541 |  |  |  | .164 |
| No | 32 |  |  |  |  |  |  |  |
| Yes | 16 |  |  |  |  |  |  |  |
| **MELD** |  |  |  | .482 |  |  |  | .270 |
| ≤ 8 | 40 |  |  |  |  |  |  |  |
| > 8 | 10 |  |  |  |  |  |  |  |
| **Child Pugh Score** |  |  |  | .589 |  |  |  | .756 |
| ≤ 5 | 37 |  |  |  |  |  |  |  |
| > 5 | 14 |  |  |  |  |  |  |  |
| **Albumin, g/l** |  |  |  | .152 |  |  |  | .228 |
| ≤ 40 | 19 |  |  |  |  |  |  |  |
| > 40 | 20 |  |  |  |  |  |  |  |
| **AST, U/l** |  |  |  | .475 |  |  |  | .673 |
| ≤ 40 | 19 |  |  |  |  |  |  |  |
| > 40 | 24 |  |  |  |  |  |  |  |
| **ALT, U/l** |  |  |  | .877 |  |  |  | .419 |
| ≤ 40 | 18 |  |  |  |  |  |  |  |
| > 40 | 17 |  |  |  |  |  |  |  |
| **GGT, U/l** |  |  |  | .757 |  |  |  | .183 |
| ≤ 100 | 17 |  |  |  |  |  |  |  |
| > 100 | 27 |  |  |  |  |  |  |  |
| **Bilirubin, mg/dl** |  |  |  | .999 |  |  |  | .657 |
| ≤ 1 | 44 |  |  |  |  |  |  |  |
| > 1 | 6 |  |  |  |  |  |  |  |
| **Alkaline phosphatase, U/l** |  |  |  | .418 |  |  |  | .670 |
| ≤ 100 | 17 |  |  |  |  |  |  |  |
| > 100 | 26 |  |  |  |  |  |  |  |
| **Platelet count, 1/nl** |  |  |  | .257 |  |  |  | .852 |
| ≤ 200 | 22 |  |  |  |  |  |  |  |
| > 200 | 28 |  |  |  |  |  |  |  |
| **Prothrombin time, %** |  |  |  | .213 |  |  |  | .791 |
| ≤ 100 | 36 |  |  |  |  |  |  |  |
| > 100 | 14 |  |  |  |  |  |  |  |
| **INR** |  |  |  | .522 |  |  |  | .241 |
| ≤ 1 | 21 |  |  |  |  |  |  |  |
| > 1; < 1.2 | 24 |  |  |  |  |  |  |  |
| ≥ 1.2 | 5 |  |  |  |  |  |  |  |
| **Creatinine, mg/dl** |  |  |  | .999 |  |  |  | .825 |
| < 1 | 32 |  |  |  |  |  |  |  |
| ≥ 1 | 18 |  |  |  |  |  |  |  |
| **Hemoglobin, g/dl** |  |  |  | .213 |  |  |  | .638 |
| ≤ 12 | 14 |  |  |  |  |  |  |  |
| > 12 | 36 |  |  |  |  |  |  |  |
| **Operative time, min** |  |  |  | **.006** |  |  |  | .190 |
| ≤ 180 | 16 | 1 |  |  |  |  |  |  |
| > 180 | 35 | 7.33 | 1.75 – 30.66 |  |  |  |  |  |
| **Blood transfusions** |  |  |  | .574 |  |  |  | .135 |
| No | 21 |  |  |  |  |  |  |  |
| Yes | 30 |  |  |  |  |  |  |  |
| **FFP** |  |  |  | n. a. |  |  |  | n. a. |
| No | 0 |  |  |  |  |  |  |  |
| Yes | 51 |  |  |  |  |  |  |  |
| **Platelet transfusion** |  |  |  | .977 |  |  |  | .324 |
| No | 49 |  |  |  |  |  |  |  |
| Yes | 2 |  |  |  |  |  |  |  |
| **Laparoscopic resection** |  |  |  | .952 |  |  |  | .505 |
| No | 43 |  |  |  |  |  |  |  |
| Yes | 8 |  |  |  |  |  |  |  |
| **Type of surgery** |  |  |  | .607 |  |  |  | .624 |
| Atypical | 15 |  |  |  |  |  |  |  |
| Segmentectomy | 6 |  |  |  |  |  |  |  |
| Bisegmentectomy | 3 |  |  |  |  |  |  |  |
| Hemihepatectomy | 14 |  |  |  |  |  |  |  |
| Extended liver resection | 12 |  |  |  |  |  |  |  |
| other | 1 |  |  |  |  |  |  |  |
| **Pringle maneuver** |  |  |  | .608 |  |  |  | .471 |
| No | 46 |  |  |  |  |  |  |  |
| Yes | 5 |  |  |  |  |  |  |  |

*Hazard ratios are shown for statistically significant variables. ALT, alanine aminotransferase; ASA, American society of anesthesiologists classification; AST, aspartate aminotransferase; BCLC, Barcelona clinical liver cancer staging system; BMI, body mass index; FFP, fresh frozen plasma; GGT, gamma glutamyltransferase; INR, international normalized ratio; MELD, model of end stage liver disease; UICC, Union for international cancer control.*
