# Supplementary material for: Intraoperative Transfusion of Fresh Frozen Plasma Predicts Morbidity Following Partial Liver Resection for Hepatocellular Carcinoma
Source: J Gastrointest Surg. 2020 Jun 3;25(5):1212–23. doi: 10.1007/s11605-020-04652-0 (PMC8096754; doi:10.1007/s11605-020-04652-0)
Supplement: Supplementary file 2 — (DOCX 19 kb) [file 11605_2020_4652_MOESM2_ESM.docx]

**Supplementary Table S2:**

Group comparison of pre- and intraoperative variables between patients with and without intraoperative FFP transfusion

| **Variables** | **FFP vs. No FFP analysis** | | |
| --- | --- | --- | --- |
|  | **FFP cohort (n=51)** | **No FFP cohort (n=70)** | **p-value** |
| **Demographics and tumor characteristics** |  |  |  |
| Gender, m/f (%) | 38 (74.5) / 13 (25.5) | 46 (65.7) / 24 (34.3) | .300 |
| Age (years) | 69 ± 8 | 65 ± 12 | .219 |
| BMI (kg/m^2^) | 27 ± 4 | 26 ± 5 | .488 |
| Portal vein embolization, n (%) | 2 (3.9) | 4 (5.7) | .654 |
| ASA, n (%) |  |  | .659 |
| I | 0 | 0 |  |
| II | 22 (43.1) | 25 (35.7) |  |
| III | 27 (52.9) | 42 (60.0) |  |
| IV | 2 (3.9) | 3 (4.3) |  |
| V |  | 0 |  |
| Milan criteria, n (%) | 13 (25.5) | 24 (34.3) | .300 |
| BCLC, n (%) |  |  | .057 |
| 0 | 0 | 3 (4.4) |  |
| A | 29 (58.0) | 41 (60.3) |  |
| B | 10 (20.0) | 19 (27.9) |  |
| C | 11 (22.0) | 5 (7.4) |  |
| D | 0 | 0 |  |
| Largest tumor diameter (mm) | 74 ± 41 | 62 ± 42 | **.035** |
| Number of nodules | 2 ± 1 | 2 ± 2 | .621 |
| Macrovascular invasion, n (%) | 16 (33.3) | 13 (18.8) | .074 |
| **Preoperative liver function** |  |  |  |
| MELD Score | 7 ± 3 | 7 ± 3 | .109 |
| Albumin (g/dl) | 39 ± 6 | 40 ± 7 | .578 |
| AST (U/l) | 60 ± 55 | 54 ± 38 | .607 |
| ALT (U/l) | 50 ± 51 | 50 ± 39 | .886 |
| GGT (U/l) | 199 ± 174 | 171 ± 187 | .153 |
| Total bilirubin (mg/dl) | 0.7 ± 0.4 | 0.6 ± 0.4 | .559 |
| Platelet count (/nl) | 234 ± 115 | 239 ± 112 | .714 |
| Alkaline Phosphatase (U/l) | 131 ± 81 | 127 ± 103 | .224 |
| Prothrombine time (%) | 94 ± 16 | 94 ± 13 | .970 |
| INR | 1.05 ± 0.12 | 1.03 ± 0.09 | .773 |
| Creatinine (mg/dl) | 1.1 ± 0.5 | 1.1 ± 1.0 | .164 |
| Haemoglobin (g/dl) | 13.2 ± 2.0 | 13.0 ± 1.9 | .456 |
| Child Pugh, n (%) |  |  | .233 |
| A | 45 (90.0) | 65 (95.6) |  |
| B | 5 (10.0) | 3 (4.4.) |  |
| C | 0 | 0 |  |
| Child Pugh score | 5 ± 1 | 5 ± 0 | .073 |
| **Operative Data** |  |  |  |
| Laparoscopic resection, n (%) | 8 (15.7) | 19 (27.1) | .135 |
| Operative time (minutes) | 230 ± 86 | 196 ± 82 | **.027** |
| Operative procedure, n (%) |  |  | .305 |
| Atypical | 15 (29.4) | 23 (32.9) |  |
| Segmentectomy | 6 (11.8) | 15 (21.4) |  |
| Bisegmentectomy | 3 (5.9) | 8 (11.4) |  |
| Hemihepatectomy | 14 (27.5) | 13 (18.6) |  |
| Extended liver resection | 12 (23.5) | 8 (11.4) |  |
| other | 1 (2.0) | 3 (4.3) |  |
| Additional procedures (RFA, etc.), n (%) | 0 | 2 (3.8) | .198 |
| Pringle maneuver, n (%) | 5 (9.8) | 2 (2.9) | .111 |
| Duration of pringle maneuver (min)* | 20 (18 – 33) | 16 (12 – n.a.) | .381 |

Data presented as mean and standard deviation if not noted otherwise. Categorical data were compared using the chi-squared test, fisher’s exact test or linear-by-linear association according to scale and number of cases. Data derived from continuous variables of different groups were compared by Mann-Whitney-U-Test. Postoperative liver failure was assessed by the 50-50-criteria. *Median and interquartile range.

*ALT, alanine aminotransferase; ASA, American society of anesthesiologists classification; AST, aspartate aminotransferase; BCLC, Barcelona clinical liver cancer staging system; BMI, body mass index; FFP, fresh frozen plasma; GGT, gamma glutamyltransferase; INR, international normalized ratio; MELD, model of end stage liver disease.*
